# Protocol for SonoSpeech Cleft Pilot: a mixed-methods pilot randomized control trial of ultrasound visual biofeedback versus standard intervention for children with cleft lip and palate

**DOI:** 10.1186/s40814-022-01051-x

**Published:** 2022-04-27

**Authors:** Joanne Cleland, Lisa Crampin, Linsay Campbell, Marie Dokovova

**Affiliations:** 1 University of Strathclyde, Glasgow, Scotland; 2 NHS Greater Glasgow and Clyde, Glasgow, Scotland

**Keywords:** Cleft lip and palate, Articulation intervention, Ultrasound visual biofeedback

## Abstract

**Background:**

Children with cleft lip and palate can continue to have problems producing clear speech after surgery. This can lead to social, emotional, and educational challenges. Typical treatment involves teaching children the correct tongue movements to produce speech sounds. This is known as articulation intervention. However, this intervention is challenging because the tongue is hidden from view and movements are difficult to see and describe. This pilot randomized control trial will try a new treatment, ultrasound visual biofeedback (U-VBF) versus standard articulatory intervention for children with cleft lip and palate, as comparison. Feasibility outcomes will be determined.

**Methods/design:**

The Sonospeech project will enroll up to 40 children with cleft lip and palate aged 4;6 to 16 in a mixed-methods randomized controlled trial with blinded assessors. Children will receive either six sessions of U-VBF or articulation intervention. The primary goals of this pilot are to assess the feasibility and inform the design of a full-scale RCT of U-VBF for children with cleft speech characteristics. This will be achieved by determining the following outcome measures: recruitment/attrition rates; measures of pre-post follow-up completion; and acceptability of the randomization and interventions to families.

**Discussion:**

Larger trials of speech interventions for children with cleft lip and palate are needed. This pilot/feasibility study will determine whether a larger randomized control trial comparing ultrasound and articulation interventions is feasible.

**Trial registration:**

ISRCTN, ISRCTN17441953. Registered 22 March 2021. See Table 2 in Appendix 1 for all items.

## Background

Cleft lip and palate (CLP) is the most common congenital craniofacial abnormality, occurring in 1 in 700 live births, depending on geographical area [1]. Problems producing intelligible speech occur in CLP, even after surgery to repair the palate, and in some children cleft speech characteristics (CSC) persist, requiring intervention from a speech and language therapist (SLT) [2]. This unintelligible speech has adverse social and educational consequences, with the speech of children with CLP rated as more likely to belong to someone who is associated with negative stereotypes about appearance and socialization [3].

Standard treatment in Scotland is articulation intervention (AI), or motor-phonetic intervention, although there is also recent research supporting linguistic-phonological therapy approaches [4]. The articulation intervention approach involves teaching children correct placement for their articulators (primarily the tongue) through verbal description and demonstration [5]. AI is a challenging intervention for both the clinician and the patient because speech movements are both difficult to see and describe, due to the main articulator, the tongue, being largely hidden from view. This problem can be circumvented by using instrumental articulatory techniques which view and measure the articulators directly.

Over the last few decades electropalatography (EPG) has dominated the literature as the instrumental technique of choice for people with cleft lip and palate [6]. EPG measures tongue-palate contact using an artificial palate with electrodes embedded in it [7]. This real-time dynamic image of tongue-palate contact can be used as a biofeedback tool to teach patients about correct placement of the tongue. However, a Cochrane review of EPG by Lee and colleagues [6] found that only one study met inclusion criteria. Despite a large number of studies using EPG, most were small n or case studies. It is likely that larger studies using EPG are sparse because of practical issues with using this tool. Each patient requires an expensive custom-made palate; moreover, this custom-made palate only fits for a limited time period due to changing dentition and planned surgeries.

In contrast, ultrasound visual biofeedback (U-VBF) is growing in popularity as biofeedback tool for children with speech disorders [8]. U-VBF holds several practical advantages over EPG: it is cheaper and does not require individualized equipment. The one-off outlay cost of an ultrasound system that is suitable for speech therapy is approximately $6,000 [9], initial outlay for EPG is similar, but in addition each patient requires an EPG plate costing between $300-$570, not including the dental impression cost [7]. Moreover, while EPG images only tongue-palate contact from the alveolar region to the boundary of the hard and soft palate, U-VBF images from near the tongue tip to the root, with pharyngeal articulations, common in CLP, clearly visible. This makes U-VBF arguably the technique of choice for CLP [10]; yet, it is relatively new to the Speech and Language Therapy clinic. This is because it is only now that ultrasound systems for measuring articulation provide fast enough frame rates at an affordable cost.

A recent study showed that ultrasound can be used to identify all of the CSC described in the instrumental literature and that ultrasound assessment has better reliability than traditional perceptual approaches [11]. However, U-VBF has to our knowledge only been used in one small study with two participants with CLP [12]. In that study the U-VBF treatment was effective for one of the children, aged 6;2, who achieved accurate production of target sounds and generalized them to untreated words. In children with other types of speech sound disorders, U-VBF shows positive outcomes for the majority of children and it is particularly useful for establishing new articulations [8]: an area of particular difficulty in CLP [5]. U-VBF is therefore potentially a useful tool for establishing new articulations in children with CLP. The aim of this study is therefore to assess the feasibility and inform the design of a full-scale RCT of U-VBF for children with cleft speech characteristics. Key objectives and success criteria for proceeding to full trial are given below. If these are met, a full-scale RCT will proceed using a similar design. Questionnaires and focus groups will provide qualitative data from patients and carers. If this points to specific improvements that can be made in the design, and they do not interfere with the aims of the large-scale study, they will be implemented.

## Methods/design

### Aims

The aim of this study is to assess the feasibility and inform the design of a full-scale RCT of U-VBF for children with cleft speech characteristics. The primary aim of U-VBF is to enable learning of new articulatory gestures (new speech sounds), with secondary aims of improving intelligibility and health-related quality of life.

### Design and setting

A single-center mixed-methods two-arm parallel group pilot randomized controlled trial design will be used. The study is a single blind pilot randomized controlled trial, with control offered U-VBF therapy at the end of the study. A qualitative study (focus group) of the acceptability of both interventions and the study design will also be undertaken. All intervention will take place at the speech and language therapy hub in the Royal Hospital for Children in Glasgow. Eligibility screens and pre- and post-intervention assessments will take place either in person in a university clinic room or via telehealth (Zoom^TM^ or Microsoft TEAMS^TM^). The roles and responsibilities are outlined in Appendix 2.

### Research questions

No definitive comparisons of the interventions will be undertaken. The feasibility of a full-scale RCT will be determined by evaluating a number of objectives against set success criteria (bulleted below) taken from a similar pilot RCT of children with speech disorders [13]:

#### Objectives

1. To determine recruitment and attrition rates.
  75% of children and their families identified agree to participate.
  75% of children allocated in each group are retained for the duration of the study.
2. To measure pre-post and follow-up outcome measure completion.
  75% of outcome measures are completed.
3. To measure within-session outcome measure completion.
  Data is reported from 75% of intervention sessions.
4. To determine acceptability of randomization to children and their families.
  75% of children and their families rate randomization as acceptable in a questionnaire.
5. To determine the acceptability of ultrasound visual biofeedback as an assessment tool (both groups) and intervention tool (U-VBF group).
  75% of children and their families rate ultrasound as an acceptable technique in a questionnaire.
  Focus group analysis contains more positive than negative themes regarding acceptability
6. To measure adherence to the treatment protocol.
  75% of sessions reach the minimum dosage of 100 trials in both treatment arms.

In addition to the quantitative objectives measuring acceptability using questionnaires, this data will be validated using focus group interviews with parents and participants who volunteer. The interviews will be analyzed inductively to identify emergent themes and deductively, using the Theoretical Framework of Acceptability [14].

## Methods

A single-center mixed-methods two-arm parallel group pilot randomized controlled trial with blinded assessors will be carried out. Cases will be stratified by three age groups (4;6–7;11; 8;0–11;11 and 12;0–16;0). A wide age-range was chosen for pragmatic reasons. That is, to both aid recruitment and reflect the typical age range treated with both types of intervention. This will also allow us to gather information about the feasibility of the treatment across different ages and use this information to inform the eligibility criteria of the large-scale study. Due to the nature of the U-VBF, therapists and patients will not be blinded to treatment allocation but the limitations of this will be mitigated by the use of an assessor, blinded to group, and evaluators, blinded to both group and treatment time point. Unblinding to the assessor or the evaluator will not be permissible and is considered unnecessary given the clinicians delivering the intervention are not blind to treatment allocation.

### Participants

#### Inclusion criteria

Children managed by the Scottish Cleft Lip and Palate service aged 4;6 to 16 will be identified and recruited when attending SLT clinics at the Royal Hospital for Children in Glasgow. Invitation letters containing the complete study information sheets will be sent to families. Inclusion criteria regarding age and cleft-type are broad to reflect current clinical practice; however, inclusion criteria for type of cleft speech characteristic are narrower to ensure children are likely to benefit from either U-VBF or the control intervention. Children are eligible if they have at least one speech error that would normally be a candidate for articulation intervention. This will be assessed by a researcher SLT who is not involved in the therapy. We aim to recruit 20 children to each arm of the trial. Recruitment will stop once the target number of participants is recruited and within two years from the beginning of the study. No interim analyses will be performed before all data is collected. In summary, the inclusion criteria are: children aged 4;6 to 16, with any oral cleft-type, who have at least one speech error which requires articulation intervention.

#### Exclusion criteria

The exclusion criteria are: an uncorrected bilateral hearing loss of greater than 30 dB (from previous reports), planned surgery within the next three months, or severe language deficit (from previous SLT reports and a receptive vocabulary standard score < 70 on the BPVS-3, [15] ). Children will be allowed to continue all other medical and speech and language interventions (for example interventions targeting language or social interaction goals may be provided by community SLTs) during the trial. We will collect information on any other speech and language interventions received during the trial.

#### Randomization

Following baseline, the children will be randomized by the Glasgow Clinical Trials Unit in a 1:1 ratio, stratified for age. Children randomized to the control arm will be offered U-VBF at the end of the trial if they still present with CSCs which are candidates for U-VBF for ethical integrity and also because previous studies have shown this improves the acceptability of a randomized trial to families [13].

### Eligibility/baseline assessments

We will screen potential participants from case-notes and invite them to attend an initial screening and baseline assessment. The person carrying it out will obtain consent, ensuring the participants and their families have had time to read, understand, and discuss the information about the study, prior to any assessment or procedures. The person carrying out this assessment will not be involved in the therapy. This assessment will be either in person or via video conferencing, with in-person preferred. Screening assessment will comprise the British Picture Vocabulary Test 3 [15] to screen for adequate receptive vocabulary. This test does not require verbal responses or assess speech production, instead participants point to responses. The screening will also include a speech assessment protocol to determine whether patients present with at least one cleft speech characteristic which would be amenable to both U-VBF and the control intervention. This assessment protocol comprises the Diagnostic Evaluation of Articulation and Phonology [16] articulation and phonology subtests and an ultrasound tongue imaging protocol designed in a previous project [11] to identify covert speech errors from consonants at all places of articulation and sentences from the GoS.SP.aSS.’98/ CAPS-A [17] (Appendix 1). Families who opt for the assessment over video-conferencing will complete the same assessments, but the ultrasound tongue imaging protocol will be replaced with an audio-perceptual assessment of the same materials^1^.

There are some limitations that may arise as a result of online video assessment, such as detecting sounds where the articulators are not visible (e.g., /k/ or /g/) or detecting high-frequency sounds (e.g., /s/ or /ʃ/), despite overall good agreement rates reported for online articulation assessments [18]. However, we retain this option for participants to address potential national and local pandemic regulations [19] during which travelling to a university for assessment is not classified as essential travel.

### Speech target selection

Children with CLP may present with multiple CSCs affecting intelligibility. We intend to select as intervention targets speech sounds which are (1) amenable to treatment with both interventions and (2) likely to have the biggest functional impact on intelligibility, based on the prevalence of the error during assessment and how frequently the target sound occurs in English minimal pairs [20]. Following the screening assessments, we will select wordlists targeting each child’s specific lingual errors from a battery. In English, lingual speech sounds (imageable with ultrasound and amenable to treatment with both interventions) are /t,d,n,r,l,s,z,∫,ʒ,tʃ,dʒ,j,k,ɡ,ŋ/ and all vowels. Children with CLP are more likely to have difficulty with anterior consonants /t,d,s/. Wordlists containing these speech sounds will also form a key outcome measure (see below). Two wordlists per error type will be selected, firstly an “untreated probe” (i.e., the words will not be used in the course of therapy, and this allows us to check for generalization of targets). Secondly a “treated probe”, containing high-frequency and functionally useful words will be used to train speech targets in the course of therapy. The wordlists contain lingual targets in increasingly complex contexts from single syllable words/pseudowords through to multi-syllabic words and sentences. Where the child has more than one error, multiple wordlists will be used; however, only one treatment target (the speech sound with the most errors) will be selected and the other errors will serve as “control segments” (i.e., speech sounds that should not improve during the course of therapy unless maturation is a factor). Wordlists will be analyzed for percentage target consonants correct (PTC). Children must score < 30PTC at baseline to be eligible for the study.

### Interventions

Both interventions will be delivered by the cleft palate specialist SLTs in the Royal Hospital for Children in Glasgow. Therapy in both treatment arms will be once per week for six sessions with each session lasting up to 45 min. The number of sessions is pragmatic in nature, reflecting current practice, and is designed to highlight initial response to both interventions. It is likely that some children in both arms of the trial may require further speech intervention in the future (after the follow-up measures are taken), and this will be provided in line with standard practice, which includes further U-VBF. Both interventions will focus on acquisition of new speech sounds. Previous studies of children with non-cleft speech disorders show that a new speech sound can be acquired within one to two sessions [21] of U-VBF for most children but that some children take four to six sessions. Target articulations will be decided individually. Both interventions begin with a pre-practice phase where the aim is to teach the child to approximate the target articulation before they can begin the practice phase where at least 100 repetitions are required for learning and generalization. The dosage of a minimum of 100 trials is set in line with [22] who report a minimum of 60 to 120 trials per session for motor-based treatments. However, we recognize that the literature on dosage required during intervention for CSCs is sparse and that there is much variation reported in the literature on articulation intervention [23]. This dosage will be measured in both interventions. In this pilot we will focus on both the pre-practice phase and the first stage of practice: acquisition of a new sound in simple contexts such as “ta, tea, toe” building to short words such as “tap, team, tore” as this is feasible within six intervention sessions. If children are super-responders (i.e., they quickly retain the new speech sound and are able to produce it in complex contexts) then the protocol will also allow us to measure this. Participants will be discontinued from the intervention if they show any adverse effects to the ultrasound treatment—in rare cases, the ultrasound gel can cause contact dermatitis [24].

### Articulation intervention (AI)

This intervention involves working on a single speech sound at a time [23]. The SLT uses modelling, demonstration, verbal description, and feedback in the pre-practice phase to teach the child the new sound at first in limited contexts and then in words and finally in conversation in the practice phase. To increase parity with U-VBF, and in line with newer theories of motor learning [25], we will standardize the in-session dosage during the practice phase to at least 100 trials, i.e., each child will be given 100 attempts to articulate their target articulation in each session.

### Ultrasound visual biofeedback (U-VBF)

This intervention is grounded in the principles of motor learning. The patient sees a real-time image of their tongue moving (see [9] for a video) and guided by the SLT uses this biofeedback to learn a new articulation, building productions to increasingly complex contexts, as in AI. Again, a minimum of 100 trials are required in the intervention. The intervention is set out in an open access manual [26] and involves using the software Sonospeech^TM^. The software has functionality to be used as an assessment and intervention tool, allowing the SLT and patient to record and playback ultrasound video with synchronized audio or to view it live. The clinicians delivering the intervention have completed training and currently use ultrasound in their clinical practice.

### Outcome measures

A blinded assessor will collect measures at baseline (-t1: pre-treatment), 2 months post-randomization (t7, to allow for any delays in referral to therapy), and 3 months post-randomization (t8) to see if any benefit is maintained. Assessment will take place in a university clinic or via video-conferencing and will be carried out by a research SLT blinded to group for primary outcome measures and by the treating SLTs for within-session measures (Table 1).

**Table 1:**
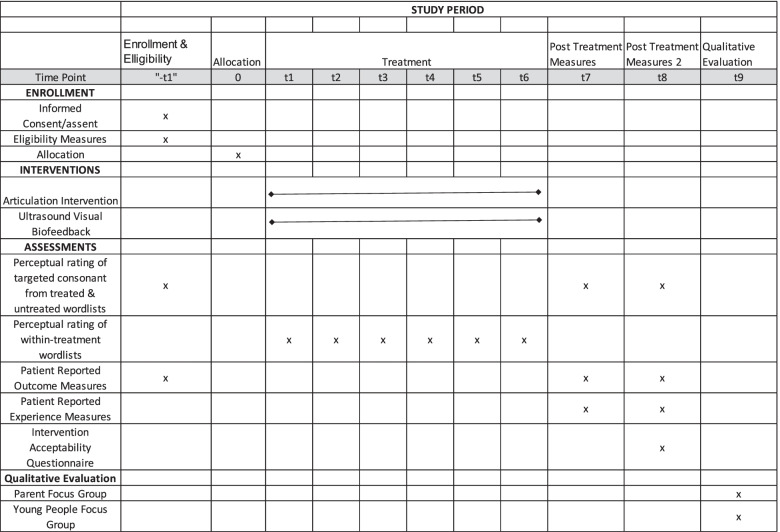
Shows the timeline for the project, including the timing of each assessment

### Within-treatment session outcomes

Previous research suggests that one of the main benefits of U-VBF may be efficiency rather than overall efficacy of treatment [8]. We will therefore measure treatment response during each session, rather than just after the course of treatment. The treating SLTs will therefore audio record short treated, and untreated word lists at every treatment session (t1–t6). From this, we will determine how quickly children achieve a new articulation as a measure of response to treatment. These will be rated at the end of the project by SLTs blinded to group.

### Candidate primary outcome measures

The key primary outcome linear measure for change in speech will be percentage target consonants correct (PTC). We will measure this at single sound level (stimulability and in /aCa/ contexts); single word level; and sentence level in treated and untreated wordlists (i.e., probes, see above section on speech target selection). All direct speech measures will be recorded with audio and where possible ultrasound tongue imaging in both groups, allowing us to perform ultrasound analysis of data from both groups.

### Patient reported outcome measures

We will use the Intelligibility in Context Scale [27] as a carer reported outcome. This short scale asks parents/carers to rate how easy to understand their child is to a variety of listeners ranging from family members to strangers. It has been shown to have a high internal reliability and construct validity [27]. Quality of life will be measured using the CLEFT-Q speech function and quality of life scales for children aged 8 and over [28]. This instrument has good content and construct validity, and good reliability, as established using a large and diverse international sample of participants [28]. We will also use the Experience of Service Questionnaire [29] to measure patient and carer satisfaction with both interventions at the end of the project. It was shown to have good construct validity and precision in measuring satisfaction with care [29]. The bespoke data collection forms, such as the participant information sheets and the consent form templates, can be found in a publicly accessible folder on the University of Strathclyde open access Knowledge Base “PURE” [30] (or via DOI 10.15129/f65343c4-7781-44fe-9b00-516d4597efac).

### Intervention acceptability measures and qualitative evaluation

Families will complete a questionnaire about the acceptability of both interventions at the end of the study. These questionnaires will also be offered to families of participants who drop out of the study. Participant retention will be promoted by dedicating time to discuss any questions and problems that may have arisen for the participant and their family at the start of each session and by offering further ultrasound or articulation intervention after the final follow-up if indicated. Parents/carers and children over 12 will be invited to join focus groups to discuss their experiences of taking part in the trial and to contribute to planning a larger trial. Focus groups will be run by the PI and an RA online using the topic guide available in a publically accessible folder available at [30] (or via DOI 10.15129/f65343c4-7781-44fe-9b00-516d4597efac). Each focus group will include up to 10 participants. Responses to the focus groups will be analyzed by an RA using thematic analysis, following the same methods employed in a similar study with children with cerebral palsy [31], that is, inductive thematic analysis.

### Analyses and statistical power

We aim to recruit 20 children to each arm of the trial. Recruitment will stop once the target number of participants is recruited and within two years from the beginning of the study. No interim analyses will be performed before all data is collected. Definitive comparisons of the interventions will not be undertaken due to the feasibility nature of the study. Details of patient screening, recruitment, retention, withdrawal, and follow-up will be summarized (see research questions above). Adherence to U-VBF will be measured according to the number of patients who complete the intervention in accordance with the treatment manual. Adherence to treatment dosage will be recorded using an intervention pro-forma in each session where the SLT records a tick mark for each trial (i.e., each time the patient attempts to produce the speech sound in treatment, this should be around 100 for both interventions). All sessions will be audio-recorded (consent permitting) and 20% of sessions will be fidelity checked for dosage and adherence to protocol by the research SLT, blinded to group allocation.

### Ultrasound/speech analysis

All of the speech measures at baseline, and follow-up will be recorded with simultaneous ultrasound in both groups by a research SLT blinded to group where possible. Our previous work showed that the addition of ultrasound to transcription increases inter-rater reliability and allows identification of covert (imperceptible to the ear alone) errors [9]. This will allow us both to calculate PTC (the primary outcome measure) with increased reliability and to perform an error analysis. Twenty-five percent of the data will also be rated by two specialist cleft SLTs (not involved in the project) trained in ultrasound-aided transcription. These SLTs will also rate the audio recordings from the within-treatment sessions (t1 to t6 in Table 1), blinded to group.

### Data management

All data will be stored in a dedicated secure shared drive managed by the University of Strathclyde where it will be automatically backed up. Hard copies of data will be kept at the hospital and the University of Strathclyde. Participants will be pseudo-anonymized during the project, with a key linking codes to actual names held securely at the university. The key will be deleted at the end of the project and data will become anonymous. Participants and their families will be informed that recordings of voice can be identifiable.

### Harms

Based on previous studies using ultrasound intervention for speech sound disorders, no harm to the participants is anticipated. In rare cases, some people may have an allergic reaction (contact dermatitis) to the gel [24]. If this is observed, the gel can be substituted with water. If water does not achieve a sufficiently good ultrasound image, then the participant may be discontinued from the trial. If any adverse consequences are observed, they will be recorded in the participant’s clinical notes and reported to the NHS ethics committee using standard procedures. The ethics committee and the university ethics committee can audit the trial at any time. The study has been granted ethics approval by the West Midlands-South Birmingham Research Ethics Committee, reference number 21/WM/0104.

### Dissemination policy

The results from this study will be published in academic journals, such as The Cleft Palate Craniofacial Journal, Clinical Linguistics and Phonetics. Additionally, results will be presented at academic and clinical conferences such as the International Congress of Cleft, Lip and Palate and Related Craniofacial Anomalies, and the conference of the Royal College of Speech and Language Therapists, where it will reach both an international and a local audience of clinicians.

The protocol, statistical code and the anonymized final numerical dataset will be made available publicly on the University of Strathclyde’s open access repository “PURE”. Protocol changes will be reported to the trial registry and the ethics committee.

## Discussion and summary

This pilot feasibility study will determine whether a full-scale randomized control trial comparing ultrasound visual biofeedback to treatment as usual (articulation intervention) for the treatment of cleft speech characteristics is feasible. Prior studies such as [21] suggest that children with CLP can tolerate ultrasound recordings and studies such as [14] suggest good retention in ultrasound intervention, with only one child in this study lost to follow-up. We therefore predict that the objectives outlined above will be met, and if this is the case a full-scale, adequately powered, multi-center RCT will be warranted. Within this planned larger RCT, we hypothesize that U-VBF will show quicker acquisition of speech sounds in error, leading to shorter time in intervention, reduced frustration for patients, and cost savings. Nevertheless, there are some potential limitations to the current study. Firstly, it is not known how acceptable randomization will be to patients and carers, and it is possible that carers will express a preference for the trial intervention, given the novelty of the technology used in U-VBF [23]. To mitigate this, patients will be offered U-VBF at the completion of the study, if their speech errors remain. A previous study trialing a similar technology-based intervention with children with Down syndrome showed that no children were lost to follow-up in either arm of the trial when this was offered [32]. A further limitation of the current study, which cannot be completely mitigated, is a potential preference from the treating clinicians for one intervention over the other, in particular the trial intervention. This could lead to increased outcomes in the trial intervention, which are not due to the visual biofeedback per se, but more to increased motivation in the intervention sessions [33]. Finally, a recent move towards more use of telehealth due to the COVID-19 pandemic, means that U-VBF is disadvantaged compared to articulation intervention, because it is a behavioral intervention which requires hardware and therefore only be can only be carried out in person, rather than over video-conferencing. It therefore currently may be more difficult to recruit to a trial which involves travel to a hospital or university.

In conclusion, this feasibility study will determine whether a larger trial comparing U-VBF and Articulation Intervention is both feasible and acceptable to patients and their carers. Notwithstanding the limitations, we predict that achieving the success objectives related to recruitment, retention, and acceptability outlined above will motivate a full scale RCT which in turn seeks to determine whether U-VBF shows improved outcomes over treatment as usual for speech disorders associated with cleft lip and palate.

## Data Availability

All data underpinning this publication are openly available from the University of Strathclyde PURE Knowledge Base at: 10.15129/f65343c4-7781-44fe-9b00-516d4597efac.

## Appendix 1

Table 2

**Table 2:** WHO trial items

Data category	Information
Primary registry and trial identifying number	ISRCTN 17441953
Date of registration in primary registry	22 March 2021
Secondary identifying numbers	
Source(s) of monetary or material support	Chief Scientist Office of Scotland
Primary sponsor	University of Strathclyde
Secondary sponsor(s)	N/A
Contact for public queries	joanne.cleland@strath.ac.uk
Contact for scientific queries	joanne.cleland@strath.ac.uk
Public title	Ultrasound visual biofeedback versus standard treatment for children with cleft lip and palate
Scientific title	SonoSpeech Cleft Pilot: a pilot randomized control trial of ultrasound visual biofeedback versus standard intervention for children with cleft lip and palate
Countries of recruitment	Scotland
Health condition(s) or problem(s) studied	Cleft lip and palate
Intervention(s)	Active comparator: ultrasound visual biofeedback
	Treatment as usual: articulation intervention
Key inclusion and exclusion criteria	Ages eligible for study: 4 to 16 years
	Sexes eligible for study: all
	Accepts healthy volunteers: no
	Inclusion criteria: speech disorders associated with cleft lip and palate
	Exclusion criteria: no spoken English; severe hearing loss; severe learning disability
Study type	Interventional
	Allocation: randomized intervention model. Parallel assignment masking: single blind (investigator, outcomes assessor)
	Primary purpose: treatment
	Phase II
Date of first enrolment	October 2021
Target sample size	40
Recruitment status	Recruiting
Primary outcome(s)	Percentage targeted consonants correct measured using speech assessment as the percentage of treated speech sounds produced correctly in words at baseline, 6 weeks, and 3 months
Key secondary outcomes	1. Patient- and carer-reported speech function and intelligibility for children aged ≥ 8 years measured using the Intelligibility in Context Scale and the CLEFT-Q speech function scale at baseline, 6 weeks, and 3 months 2. Patient- and carer-reported quality of life for children aged ≥ 8 years measured using the CLEFT-Q quality of life scale at baseline, 6 weeks, and 3 months 3. Patient and carer satisfaction with both interventions measured using the Experience of Service Questionnaire at 6 weeks, and 3 months

## Appendix 2

### Roles and Responsibilities

#### Principal Investigator (Joanne Cleland)

- Design and conduct of Sonospeech
- Preparation of protocol and revisions
- Preparation of participant information sheets and consent forms
- Organizing steering committee meetings
- Publication of study reports

Steering Committee

- See title page for all members
- Agreement of final protocol
- All lead investigators will be steering committee members
- Recruitment of patients and liaising with principle investigator
- Reviewing progress of study and if necessary agreeing changes to the protocol or personnel

Trail Management Committee

- Principle Investigator and Research Speech and Language Therapist
- Study planning
- Organization of steering committee meetings
- Ethical applications
- Provide annual report to ethics committee
- Reporting of serious adverse events
- Budget administration and contractual issues
- Data checking and verification
- Randomization, in collaboration with the clinical trials unit

Data Manager

- Research Speech and language therapist
- Maintenance of data entry and data storage

## Footnotes

1 The assessment sessions are not part of core clinical care and therefore we will offer these over video-conferencing to comply with any current local COVID restrictions under which travelling for research assessments may not be an “essential journey”.
